# Targeting Rad50 sensitizes human nasopharyngeal carcinoma cells to radiotherapy

**DOI:** 10.1186/s12885-016-2190-8

**Published:** 2016-03-07

**Authors:** Lihong Chang, Jiancong Huang, Kai Wang, Jingjia Li, Ruicheng Yan, Ling Zhu, Jin Ye, Xifu Wu, Shimin Zhuang, Daqing Li, Gehua Zhang

**Affiliations:** Department of Otolaryngology-Head & Neck Surgery, The Third Affiliated Hospital, Sun Yat-sen University, NO.600 Tianhe Road, Guangzhou, 510630 China; Department of Otorhinolaryngology-Head & Neck Surgery, The First People’s Hospital of Foshan, Cancheng District, NO.81 Lingnan Bei Road, Foshan, 528000 China; Department of Otolaryngology-Head & Neck Surgery, Zengcheng District People’s Hospital of Guangzhou (Boji-Affiliated Hospital of Sun Yat-sen University), Zengcheng District, NO.1 Guangming Dong Road, Guangzhou, 511300 China; Department of Otolaryngology-Head & Neck Surgery, Nanhai Maternity and Child Healthcare Hospital, Nanhai District, NO.6 Guiping Xi Road, Foshan, 528000 China; Department of Otolaryngology-Head & Neck Surgery, The Sixth Affiliated Hospital of Sun Yat-sen University, NO.26 Yuancun Erheng Road, Guangzhou, 510655 China; Department of Otorhinolaryngology-Head & Neck Surgery, University of Pennsylvania School of Medicine, Philadelphia, PA 19104 USA

**Keywords:** Nasopharyngeal carcinoma, Radiosensitization, MRN complex, Rad50

## Abstract

**Background:**

The Mre11-Rad50-Nbs1 (MRN) complex is well known for its crucial role in initiating DNA double strand breaks (DSBs) repair pathways to resistant irradiation (IR) injury and thus facilitating radioresistance which severely reduces radiocurability of nasopharyngeal cancer (NPC). Targeting native cellular MRN function would sensitize NPC cells to IR.

**Methods:**

A recombinant adenovirus containing a mutant Rad50 gene (Ad-RAD50) expressing Rad50 zinc hook domain but lacking the ATPase domain and the Mre11 interaction domain was constructed to disrupt native cellular MRN functions. The effects of Ad-RAD50 on the MRN functions were assessed in NPC cells lines using western blot, co-immunoprecipitation and confocal microscopy analyses. The increased radiosensitivity of transient Ad-RAD50 to IR was examined in NPC cells, including MTT assay, colony formation. The molecular mechanisms of radiosensitization were confirmed by neutral comet assay and western bolts. Nude mice subcutaneous injection, tumor growth curve and TUNEL assay were used to evaluate tumor regression and apoptosis in vivo.

**Results:**

Rad50 is remarkably upregulated in NPC cells after IR, implying the critical role of Rad50 in MRN functions. The transient expression of this mutant Rad50 decreased the levels of native cellular Rad50, Mre11 and Nbs1, weakened the interactions among these proteins, abrogated the G2/M arrest induced by DSBs and reduced the DNA repair ability in NPC cells. A combination of IR and mutant RAD50 therapy produced significant tumor cytotoxicity in vitro, with a corresponding increase in DNA damage, prevented proliferation and cell viability. Furthermore, Ad-RAD50 sensitized NPC cells to IR by causing dramatic tumor regression and inducing apoptosis in vivo.

**Conclusion:**

Our findings define a novel therapeutic approach to NPC radiosensitization via targeted native cellular Rad50 disruption.

**Electronic supplementary material:**

The online version of this article (doi:10.1186/s12885-016-2190-8) contains supplementary material, which is available to authorized users.

## Background

Although representing only approximately 0.7 % of the global cancer burden [1], nasopharyngeal cancer (NPC) is the leading cancer in the South East Asia and Southern China (30.94/100,000 in males and 13.00/100,000 in females) [2], particularly in Guangdong province, Hong Kong, Philippines and Thailand [1]. Radiotherapy represents the primary treatment for early-stage NPC; The 5-year overall survival rate (OS) is at least 85.8 % for the patients with early-stage NPC [3, 4] but less than 60 % for advanced NPC after radiotherapy alone [5]. Improving the radiotherapy dose alone would affect the anatomic proximity of crucial structures and reduce the life quantity [6]. NPC patients who exhibit radioresistance are a key population that contributes to the observed poor outcomes. Improving the sensitization of NPC to radiotherapy represents a useful solution.

The predominant damage induced by irradiation (IR) is DNA double-strand breaks (DSBs), however, tumor cells exhibit a high capacity to repair DNA DSBs. Thus, the molecules or proteins responsible for DNA repair following radiotherapy are emerging as potential therapeutic targets for sensitizing cancer cells to radiotherapy and protecting surrounding normal tissues.

The Mre11-Rad50-Nbs1 complex (MRN complex) facilitates the following two DSBs repair pathways in humans: homologous recombination repair (HR) and non-homologous end joining (NHEJ). This complex plays crucial roles, including the detection of DSBs and subsequent signaling, such as the activation of ATM kinase (a key DSBs signaling protein), cell cycle regulation and telomere maintenance [7, 8]. The role of the MRN complex in the response to DSBs as well as its requirement for cellular survival makes it a potential target for sensitizing cancer cells to therapy. Rhee *et al*. constructed recombinant adenoviruses to disrupt Nbs1 function and enhance the radiosensitivity of head and neck tumors [9]. A previous study also found that the disruption of Rad50 function enhanced the chemosensitivity of head and neck cancer [10].

The human RAD50 (hRAD50) gene is located at chromosome 5q31 and encodes the 153 kDa Rad50 protein. It is an ATP-modulated DNA crosslinker that consists of the following three important domains: the bipartite ATP-binding cassette (ABC) domain, the Mre11 interaction site, and the zinc hook region. As a key component of the Mre11_2_/Rad50_2_ heterotetramer, hRAD50 is the essential for the formation of functional MRN complex. The ABC domain of Rad50 exhibits both the ATPase and adenylate kinase activity that are essential for all the known functions of the MRN complex [11, 12]. ATP binding to the ABC domain induces a conformational switch of the Rad50 protein, increasing the affinity for its substrate, promotes the Rad50-mediated regulation of Mre11 exonuclease activity [13–15], and allows the zinc hook region of Rad50 to form a protruding end to link another opposing Rad50 protein [16]. During DSBs repair, whether through NHEJ or HR, Mre11 and Rad50 form functional Mre11_2_/Rad50_2_ heterotetramers that bind to opposing DNA ends or to sister chromatids and are linked to the other heterotetramers via dimerization of the Rad50 hook domains to initial DSBs repair [16].

Given the known crucial roles of Rad50, we sought to use a recombinant adenovirus construct containing a mutant Rad50 gene, which consists only of the zinc hook region, to abrogate the formation of the Mre11_2_/Rad50_2_ heterotetramer, impair DSBs repair capacity, and disrupt MRN function and radiosensitize NPCs.

## Methods

### Cells culture

The CNE1 and 5–8 F human nasopharyngeal carcinoma cell lines were cultured in RPMI 1640 media supplemented with 10 % fetal bovine serum and 1 % penicillin-streptomycin at 37 °C in 5 % CO_2_. No ethics approval was required for the use of human cell lines in this study.

### Recombinant adenoviral infection and IR treatment

A recombinant adenovirus (Ad-RAD50) containing a fragment of the RAD50 hook located at the hinge domain of the wild-type Rad50 gene which express 13 kDa mutant Rad50 protein and green fluorescence report gene GFP was constructed in a previous study [17]. Ad-EGFP without a fragment of the RAD50 hook was used as the empty control adenovirus (Invitrogen Trading (shanghai) Co. Ltd.). Cells were infected with recombinant Ad-RAD50 at a corresponding MOI 50, 25 for CNE1 and 5–8 F for 4 h at 37 °C, followed by replacement with fresh 1640 media. The infected cells present green fluorescence and 13 kDa mutant Rad50 proteins indicated the successfully infection. The MOI of empty control adenovirus was 100.

For the cells treated with Ad-RAD50 infection combined with IR, the cells were infected with Ad-RAD50 for 4 h, followed by replacement with fresh 1640 media for 24 h, and then were treated with IR. The 4Gy IR was applied in this study. The survival rate of the cells treated with 4Gy IR exhibited a significant growth inhibition relative to the control-treated cells after 6 days, as indicated by MTT assay. The cells exhibited a survival rate of 0.59. So, all treatments except for colony formation were used 4Gy as the dose of IR. All assessments for cells treated with IR or Ad-RAD50 combining with IR were conducted at 24 h after IR.

### Western blot analysis

Total protein was extracted from cells with different treatments using total Protein Extraction Kit (KGP2100). After boiling in SDS sample buffer, the supernatant were analyzed on 10 % SDS-PAGE gels followed by transfer to 0.45 μm PVDF membranes (Millipore, USA). Nonspecific binding sites were blocked using 5 % nonfat dried milk in 1 × TBST buffer (10 mM Tris–HCl buffer at pH 7.5, 125 mM NaCl, and 0.1 % Tween-20). The membranes were incubated with different primary antibodies overnight at 4 °C, respectively (polyclonal rabbit anti-human Mre11 (ab33125,Abcam, UK), polyclonal rabbit anti-human Rad50 (ab89, Abcam, UK), polyclonal rabbit anti-human Nbs1 (ab32074, Novus, USA), monoclonal rabbit anti-human Cdc25C (phospho S216) antibody (ab32051, Abcam, UK), polyclonal rabbit anti-human-CDK1 (phospho Y15) antibody (ab47594, Abcam, UK) and polyclonal rabbit anti-human GAPDH (10494-1-AP, ProteinTech, USA). The secondary antibody was polyclonal goat anti-rabbit (CW0103,CWBIO, China). For all the Western blot experiments, the primary antibodies were used at a concentration of 1:1,000 ~ 1:3,000, and the secondary antibody was used at a concentration of 1:3,000. A pre-stained standard marker (Thermo, USA) was used for the estimation of protein molecular weight. The FluorChem M System (protein simple, Alpha Innotech Corp.) was used to visualize the protein bands and capture images of the gels. Image J software was used to analyze and quantify the densitys of the Western blot bands.

### Coimmunoprecipitation

Coimmunoprecipitation and immunoblot experiments were conducted to verify the binding interactions between mutant Rad50 and the MRN complex. CNE1 cells were either untreated or infected with Ad-RAD50, twenty-four hours after plating into 150 mm culture dishes, followed by replacement with fresh 1640 media. At 24 h after infection, a subset of cells from the untreated and Ad-RAD50 infected groups were subjected to 4Gy IR. At 24 h after IR, the cells were washed with cold 1 × PBS, scraped into cell lysis buffer for Western and IP (Beyotime, China) for 5 s, and gently transferred into centrifuge tubes and centrifuged at 1,2000 rpm for 5 min at 4 °C. Lysate preclearing was performed by rolling with BioepitopeR protein A + G agarose (Santa Cruz, USA) at 4 °C on a microcentrifuge tube roller apparatus for 1 h, followed by centrifugation at 1,2000 rpm for 1 min at 4 °C. The supernatants were retrieved and quantified using a BCA assay (KEYGEN, Biotech, China). Next, the cleared lysates were incubated with polyclonal rabbit anti-human Mre11 antibody (Abcam, USA) at a final concentration of ~2 μg Ab per 500 μg lysate, with added ZnCl_2_ to a final concentration of 100 μM and rotated overnight at 4 °C. Immune complexes were then precipitated by rolling the lysates with BioepitopeR protein A + G agarose (Santa Cruz, USA) overnight. The samples were centrifuged at 12,000 rpm for 1 min at 4 °C. Immunoprecipitates bound to the beads were washed 3 times with cell lysis buffer and were added 50 μL of 2× loading buffer. The supernatant was used for immunoblot analysis as described above.

### Confocal microscopy

CNE1 cells were plated at a concentration of 7 × 10^5^ cells/plate in 35-mm glass bottom culture plates with 15-mm glass bottoms for confocal microscopy (NEST Biotechnology Co., LTD., China) and allowed to adhere overnight. Next, the cells were either untreated or treated with Ad-RAD50, followed by replacement with fresh 1640 media after infection for 4 h at 37 °C. Adenoviral transgene induction was confirmed by visualizing GFP expression by fluorescence microscopy at 12 h after infection. At 24 h after infection, a subset of cells was subjected to 4Gy IR. Each dish was configured as follows: control cells (dish 1, 5, 9, 13), 4Gy-treated cells (dish 2, 6, 10, 14), Ad-RAD50–infected cells (dish 3, 7, 11, 15), and Ad-RAD50 with 4Gy-treated cells dish 4, 8, 12, 16). After treatments with Ad-RAD50 infection and/or IR, dishes 1–4 were treated with mouse monoclonal anti-human Rad50 (ab89, Abcam) and rabbit polyclonal anti-human Mre11 (Abcam) primary antibodies. Donkey anti–rabbit IgG Antibodies Alexa Fluor 546 and donkey anti–mouse IgG Antibodies Alexa Fluor 488 (Invitrogen) were used as second antibodies. Dishes 5–8 were treated with mouse monoclonal anti-human Rad50 and rabbit polyclonal anti-human Rad50 (zinc hook region) (NB100-1487, Novus Biological, USA) primary antibodies, followed by donkey anti–rabbit IgG Antibodies Fluor 546 and donkey anti–mouse IgG Antibodies Fluor 647 secondary anti-bodies. Dishes 9–12 were not treated with any antibodies and served as controls. Dishes 13–16 were treated only with secondary antibodies. The cell nuclei were visualized by confocal microscope at × 63 magnification. Images were captured; image pro plus software 6.0 was used to analyze signal co-localization.

### Neutral comet assay

The neutral comet assay was used to quantify DNA DSBs. CNE1 and 5–8 F cells that were not treated and all cells infected with empty virus served as controls. The other treatment groups were 4Gy IR alone, Ad-RAD50 infection alone, Ad-RAD50 with 4Gy IR and empty-Ad with 4Gy IR. All cells were grown as a monolayer in 6-well tissue culture plates and then infected with either Ad-RAD50 or empty-Ad over a 4-h period at 37 °C, followed by replacement with fresh 1640 media. A subset of the cells was subjected to with 4Gy IR. The neutral comet assay was performed at 0, 24 and 48 h after IR treatments, according to the manufacturer’s protocols (Trevigen, USA). The slides were viewed at × 40 magnifications using a microscope. Images of 30 randomly selected cells per samples group were acquired. Comet measurement and quantitative analysis were performed using CASP software. The median tail moments (MTM) were used to quantitate the extent of DNA DSBs.

### Cell growth and survival test

Three samples of 3 × 10^3^ log phase cells were plated in 96-well cell culture plates. The cells were incubated with Ad-RAD or empty-Ad (MOI 50, 25 for CNE1 and 5–8 F respectively) for 4 h at 37 °C. The cells were then cultured with fresh 1640 media for 24 h after viral infection. Next, a subset of the CNE1 and 5–8 F were subjected to 4Gy IR. After treatments, cell growth over 6 days was assessed by MTT assay.

### Colony formation assay

CNE1 and 5–8 F cells were plated at a concentration of 5 × 10^4^ in 60-mm tissue culture dishes and allowed to adhere overnight. Cells were untreated or infected with Ad-RAD50 or Ad-EGFP for 4 h at 37 °C, and then cultured with fresh 1640 media. Adenoviral transgene induction was confirmed by visualizing GFP expression under fluorescence microscopy 12 h after infection. Cells were transferred to 6-wells culture plates (corning) at 70, 70, 140, 350, 1050, 3150 cells per well and allowed to adhere overnight, and then were treated with 0, 1, 2, 4, 6, 8Gy, respectively. After 10 days, the cells were fixed with 4 % paraformaldehyde, stained with Giemsa stain kit (DM0002, LEAGE, Beijing Leagene Biotechnology Co., Ltd., China), and scored by counting colonies under an inverted microscope, using the standard definition that a colony consists of 50 or more cells. Graph pad prism 5.0 was used to fit surviving fraction curve using the equation: Y = 1 ‐ (1 ‐ exp(‐ k * x))^N^.

### Ad-RAD50 treatment of NPC xenografts

Thirty nude mice were randomized into six groups of five mice each. The right dorsal flank of each mouse was injected subcutaneously with 3 × 10^6^ CNE1 cells. After the establishment of palpable tumors, mouse body weight and external tumor volume were determined regularly. External tumor diameter was measured using digital calipers, and tumor volume was calculated using the formula A^2^ × B × 0.5, where A represents the smallest diameter and B equals the largest diameter. Eight days after tumor injection, the average tumor volume was approximately 200 mm^3^. All the mice were anesthetized with 0.1 ml 4 % chloral hydrate per 10 g via intraperitoneal injection and divided into 6 groups (Control, Ad-EGFP infection, Ad-RAD50 infection, IR, IR combined with Ad-EGFP infection, and IR combined with Ad-RAD50 infection). These six intervention groups were intratumoral PBS buffer (50 μL volume), intratumoral Ad-RAD50 (8 × 10^8^ PFUs in 50 μL volume), intratumoral Ad-EGFP (8 × 10^8^ PFUs in 50 μL volume), respectively. Twenty-four hour after intratumoral injection, mice of corresponding groups were anesthetized and suffered 6Gy IR treatment. The internal tumor volume was measured regularly until the tumors of controls reached nearly 2,000 mm^3^ at 14th day after treatments. The animal use protocol has been reviewed and approved by the Animal Ethical and Welfare Committee (AEWC). The Approved No. is IACUC-DB-15-0304.

### Quantification of apoptosis in NPC tumors

Tumors were harvested from mice after size measurement. Harvested tumors were fixed in 10 % neutral formalin, embedded in paraffin, and sectioned into 5-μM thick samples. TUNEL apoptosis staining using TUNEL APOPTOSIS DET KIT (Millipore, America) was performed according to manufacturer’s instructions. After staining, five tumor specimens were randomly selected from each treatment group. Ten × 40 high-powered fields from each specimen were randomly selected. Images were acquired using the imaging system described above. The percentage of apoptosis cells in each high-powered field was calculated.

### Statistics

Statistical analysis was performed using SPSS16.0. The two-tailed Student’s t test for independent samples was used for the analysis of all the data. *P* < 0.05 were considered statistically significant.

## Results

### Wild-type Rad50 is upregulated in NPC cells following IR

**Fig. 1:**
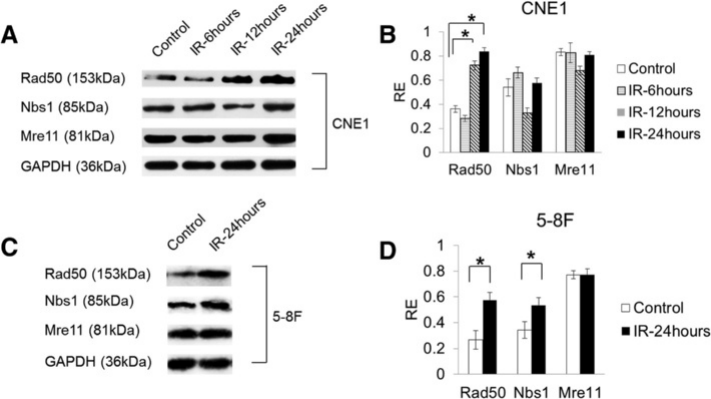
The expression of MRN complex components in NPC cells. **a** and **c** The western blot of MRN complex proteins in CNE1 and 5–8 F after IR, respectively. **b** and **d** The expression analysis of western blot in CNE1 and 5–8 F after IR, respectively. Wild-type Rad50 was upregulated at 12 and 24 h after 4Gy IR treatment in CNE1 cells and was upregulated at 24 hours after 4Gy IR treatment in 5–8 F cells. Wild-type Nbs1 was found to be upregulated in 5–8 F cells (*P* < 0.05). * *P* < 0.05

To avoid the influence of mutations and elucidate the contributions of IR induced DNA-damage, we confirmed the expression of wild-type MRN complex proteins in CNE1 and 5–8 F cells after IR. As shown in Fig. 1, after treatment of the cells with 4Gy IR, the Rad50 protein was upregulated in the two cell lines. The expression of Rad50 peaked at 24 h after IR of CNE1 cells. The expression levels of Nbs1 did not exhibit significant changes in CNE1 cells but were upregulated in 5–8 F cells, respectively (*P* < 0.05 for both).

### Expression of the mutant Rad50 disturbed the MRN complex formation

**Fig. 2:**
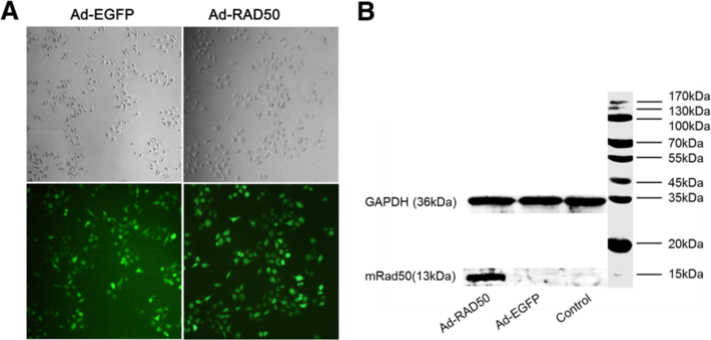
Ad-RAD50 infection and the expression of mutant Rad50 in NPC cells. **a** The successful infection of Ad-EGFP and Ad-RAD50 in CNE1 cells. **b** The expression of mutant Rad50 protein in the Ad-RAD50 infected CNE1 cell

**Fig. 3:**
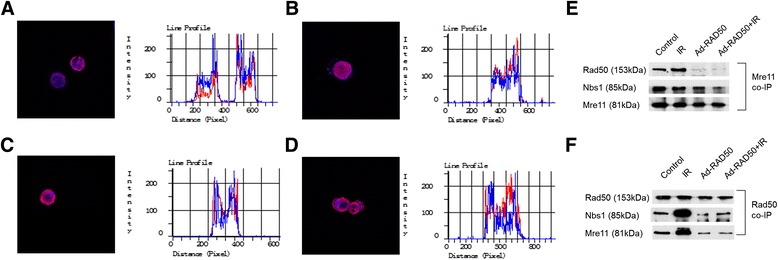
The binding interaction between Rad50 and Mre11/mutant Rad50. **a** and **b** are the confocal assays of Ad-RAD50 infected CNE1 cells, **c** and **d** the confocal assays of Ad-RAD50-treated CNE1 cells after IR. In (**a**) and (**c**), the overlap of the signals corresponds to wild-type Rad50 (*blue*) and Mre11 (*red*). In (**b**) and (**d**), the overlap of the signals corresponds to wild-type Rad50 (*blue*) and mutant Rad50 (*red*), which suggests a binding interaction between these proteins. **e** and **f** The co-IP of wild-type Mre11 and Rad50 in CNE1 cells. The Ad-RAD50-treated cells demonstrated significant inhibition of the interaction with wild-type Mre11 with Rad50 and Nbs1. Cells with Ad-EGFP treatments were used as controls

We hypothesized that the targeting of Rad50 and the disruption of its interaction with the other two MRN complex proteins would reduce radioresistance. To test this hypothesis, we used an adenoviral vector established in our previous study to infect the NPC cells. The adenoviral vector contains a gene sequence encoding a fragment of the Rad50 zinc hook region (Ad-RAD50), which completely lacks the regions that encodes the ATPase regions and Mre11 interaction sites [17]. As shown in Fig. 2, Adenoviral transgene induction in CNE1 infected with Ad-RAD50 (MOI = 50) was confirmed by visualizing green report gene expression under fluorescence microscopy 12 h after infection. The expression of the 13-kDa mutant Rad50 protein was detected by western blot in CNE1 cells infected with Ad-RAD50. This truncated form of Rad50 only possesses the hook domain, with which endogenous wild-type Rad50 protein engages in zinc-mediated dimerization, forming a single Mre11_2_/Rad50_2_ heterotetramer bound to mutant Rad50. Because this mutant Rad50 adenoviral vector lacks the Mre11 interaction site, it would abrogate the assembly of a second, functional Mre112/Rad50_2_ heterotetramer on the opposing DNA strand or sister chromatid. Furthermore, the impairment of Mre11 and Rad50 interaction elicited a subsequent effect on Nbs1. In this study, we performed a series of confocal microscopy experiments to demonstrate these binding interactions. Cells treated with Ad-RAD50 infection, and Ad-RAD50 infection plus 4Gy IR exhibited co-localization of the signals representing wild-type Rad50 (blue) and Mre11 (red), suggesting the interaction between these two proteins (Fig. 3-a, -b). The co-localization of the signals representing mutant Rad50 (red) and wild-type Rad50 (blue) confirmed the interaction between these two proteins in the cells infected with Ad-RAD50 (Fig. 3-c and -d). A series of coimmunoprecipitation experiments was used to confirm our microscopy findings. The results confirmed the binding interaction between Mre11 and wild-type Rad50 proteins based on the coimmunoprecipitation of the wild-type Rad50 protein using an anti-human Mre11 antibody (Fig. 3-e) and anti-human Rad50 antibody (Fig. 3-f) in all four groups. The Ad-RAD50 treatment significantly decreased the interaction between wild-type Rad50 and Mre11, Mre11 and Nbs1 (Fig. 3-e, -f). We confirmed that the mutant Rad50 interacted with wild-type Rad50, thus weakening the interaction between wild-type Rad50 and Mre11 and abrogating MRN complex formation.

### Ad-RAD50 infection downregulated MRN complex proteins

**Fig. 4:**
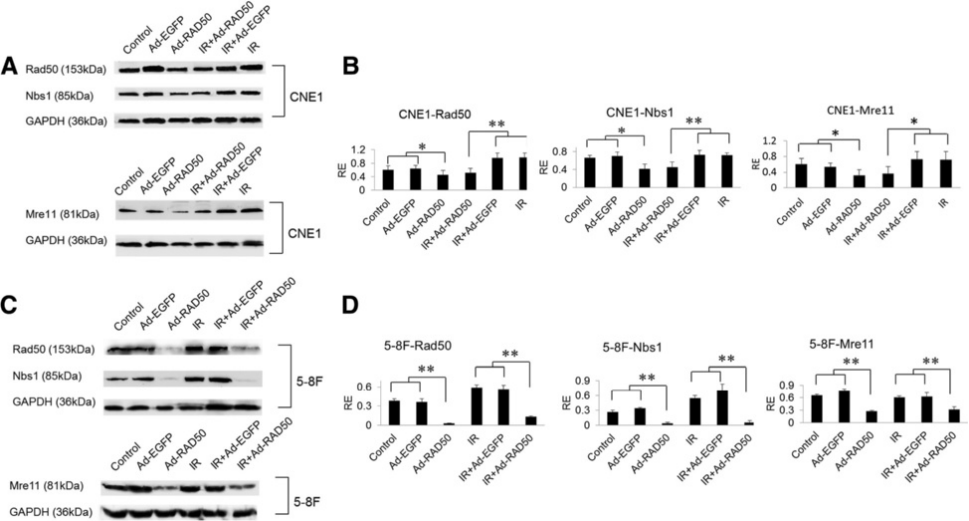
Infection of Ad-Rad50 downregulated the MRN proteins in NPC cells. **a** and **b** are the MRN complex proteins in CNE1 infection with Ad-RAD50 combing with 4Gy IR after 24 h; **c** and **d** are the corresponding results in 5–8 F cells. Downregulation of wild-type Mre11, Rad50, and Nbs1 proteins was observed in CNE1 cells or 5–8 F cells treated with Ad-RAD50 infection compared to uninfected and Ad-EGFP infected controls, combing with IR or not (the mean optical density of the Western blot bands ± SEM is shown, * *P* < 0.05, ** *P* < 0.01)

Our data indicated that a second Mre11_2_/Rad50_2_ heterotetramer could not form proximal to the mutant Rad50 protein. We performed further western blot analysis to determine whether the mutant Rad50 protein would induce a dominant-negative downregulation of the MRN complex by sequestering the wild-type Mre11_2_/Rad50_2_ heterotetramer. The infection of Ad-RAD50 induced a downregulation of the wild-type Rad50 protein in CNE1 cells and 5–8 F cells (Fig. 4). At 24 h after infection with Ad-RAD50, the Mre11 and Nbs1 proteins were also significantly downregulated in cells treated with Ad-RAD50 alone or combing with 4Gy IR. These results confirmed our hypothesis that the disruption of wild-type Rad50 function by the mutant Rad50 adenoviral vector influences the expression of the other MRN complex proteins, which might be attributable for the reduction in the DSBs repair capacity of the NPC cells.

### Mutant Rad50 enhances the IR cytotoxicity and IR sensitivity

**Fig. 5:**
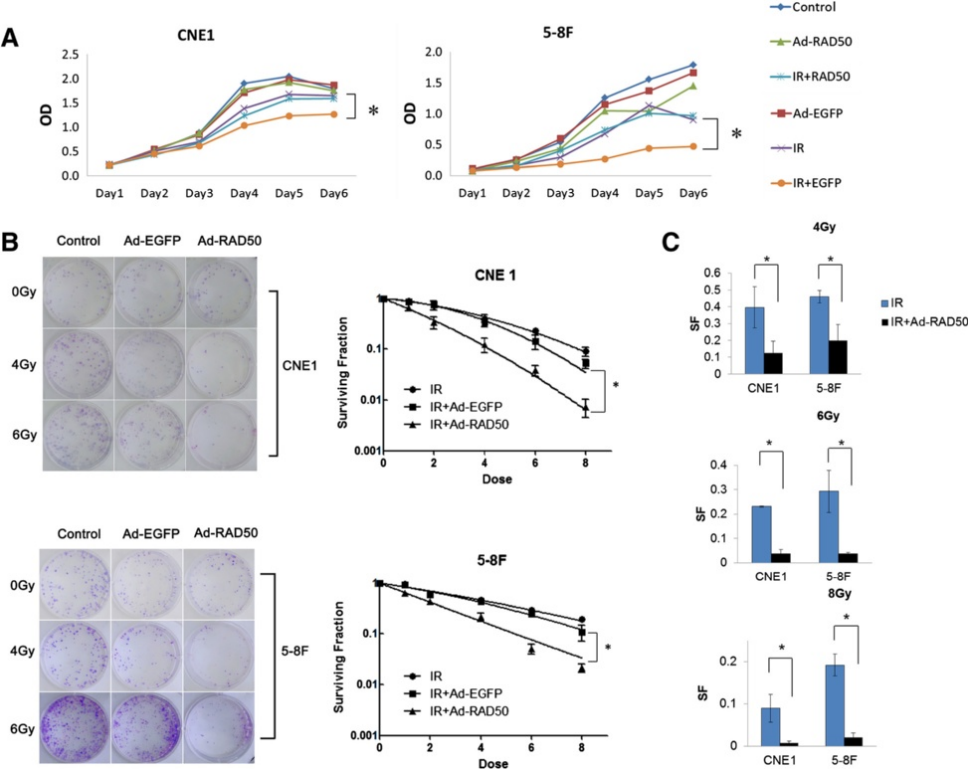
Ad-RAD50 enhanced the IR-induced cell growth suppression and IR sensitivity. **a** Cell growth suppression was measured by the MTT assay. NPC cells treated with Ad-RAD50 and 4Gy IR combination exhibited significant persistent proliferative suppression compared to all the other treatments. **b** and **c** Colony formation assay showed that Ad-RAD50 infection inhibited the cells survival fractions (* *P* < 0.05, ** *P* < 0.01)

We used a 3-(4, 5-dimethylthiazol-2-yl)-2, 5-diphenyltetrazolium bromide (MTT) cell growth assay to measure the growth of the CNE1 and 5–8 F NPC cells. Each cell line was divided into the following six groups: no treatment control, Ad-EGFP empty control virus-treated, Ad-RAD50-treated, 4Gy IR-treated, Ad-EGFP- and 4Gy IR-co-treated, and Ad-RAD50- and 4Gy IR-treated. The adenoviral vectors were introduced at an MOI of 50 for CNE1 and 25 for 5–8 F. Control-, Ad-RAD50- and Ad-EGFP-treated cells exhibited relative logarithmic growth. 4Gy IR reduced cell growth relative to the controls of CNE1 and 5–8 F cells. Treatment with Ad-RAD50 plus 4Gy IR enhanced the reduction in the growth of these NPC cells compared to IR alone. At day 6 of treatment, relative to the control, 4Gy IR alone and Ad-RAD50 alone treatments, treatment with Ad-RAD50 and IR combination therapy produced 24.9, 19.8 and 27.9 % reductions in the density of the CNE1 cells, 81.0, 59.1 and 75.7 % decreases in the number of 5–8 F cells (Fig. 5-a), respectively. Therefore, the expression of the mutant Rad50 transgene potentiated the cytotoxic effects of radiation in NPC.

We further performed a colony formation assay to test the effect of Ad-RAD50 combing with IR on NPC cells’ proliferation. As expected, Ad-RAD50 infection significantly increased IR induced inhibition the capacities of CNE1 and 5–8 F cells’ colony formation (as Fig. 5-b shown). Relative to the IR alone, combination Ad-RAD50 with 4Gy, 6Gy, 8Gy IR produced corresponding 3.193 (SF = 0.396 vs. 0.124), 5.949 (SF = 0.232 vs. 0.039) and 12.429 (SF = 0.087 vs. 0.007) fold reductions in the survival fraction (SF) of the CNE1 cells (*P* < 0.01), and corresponding 2.300 (SF = 0.460 vs. 0.200), 7.919 (SF = 0.293 vs. 0.037) and 9.190 (SF = 0.193 vs. 0.021) fold reductions in the survival fraction (SF) of the 5–8 F cells (*P* < 0.01) (Fig. 5-c).

### Enhanced cytotoxicity is associated abrogated DNA DSBs induced G2/M arrest and subsequently increased DNA DSBs

**Fig. 6:**
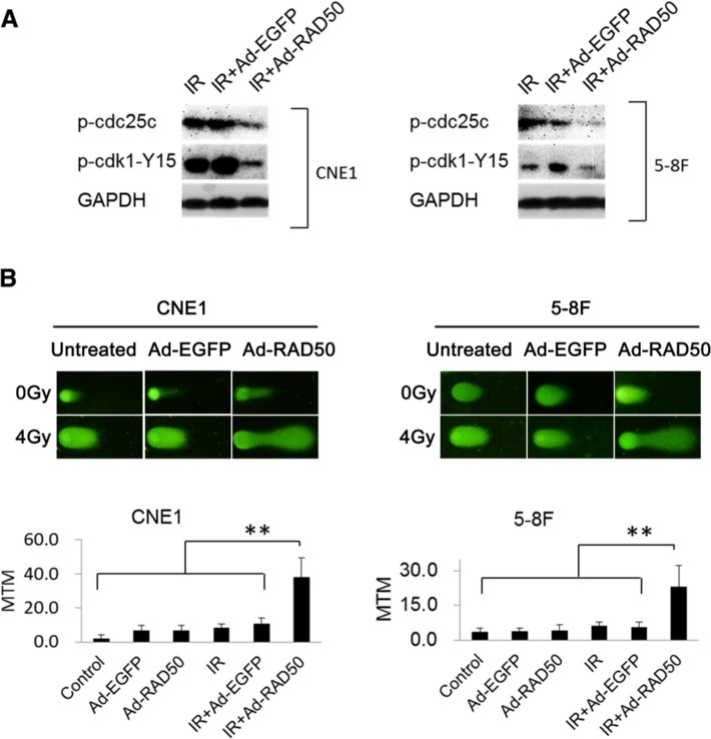
Ad-RAD50 abrogated the DSBs-induced G2/M arrest and enhances IR-induced DNA damage. **a** Ad-RAD50 infection combining with IR decreased the p-cdc25c and p-cdk1-Y15 expression compared with IR alone or Ad-EGFP infection combining with IR. **b** DNA DSBs in CNE1 and 5–8 F cells detected by neutral comet assay at 24 h after IR, respectively. Original magnification: ×40. Damaged and fragmented DNA migrates toward the anode, producing a comet tail. The mean olive tail movements (MTMs) measured by using CASP software. MTM values ± SEM are shown. Ad-RAD50 combined with 4Gy IR significantly increased DSBs compared to all the other groups (* *P* < 0.05, ** *P* < 0.01)

The key cytotoxic effect elicited by IR is DNA DSBs. One of the mainly DSBs-induced responses is G2/M arrest. Cdc25c phosphorylation and the downstream cdk1/cyclinB kinase are pivotal in regulating G2/M transition. In this study, Ad-RAD50 combined significantly decreased the phosphorylation of cdc25c and cdk1, which directly contributed to the G2/M transition and thus reduced the DSBs repair times (Fig. 6-a). We hypothesized that the infection of Ad-RAD50 would reduce the DSBs repair capacity of the NPC cells, thereby resulting in enhanced cytotoxicity in cells treated with combined Ad-RAD50 infection and IR, and this reduced DSBs repair capacity would be the result of the abrogation of the G2/M arrest.

The CNE1and 5–8 F cells were assessed for DNA DSBs using the neutral comet assay. In CNE1 cells, we detected the MTM values for cells with Ad-EGFP, Ad-RAD50 or not at 0, 24 and 48 h after 4Gy IR. We found that the difference between different treatments was obvious at 24 h after IR (Additional file 1: Figure S1). So, the further analysis of the MTM values for 5–8 F cell lines were evaluated among the different groups at 24 h after IR. As shown in Fig. 6-b, in three cell lines, IR alone increased the MTM values relative to the control treatment (8.461 vs. 2.059, *P* = 7.2E-17, 6.217vs. 3.522, *P* = 1.26E-9 in the CNE1 and 5–8 F cells). Ad-EGFP or Ad-RAD50 alone resulted in weak increased MTM values compared to the control treatment (6.705 and 6.965 vs. 2.059 in CNE1, 3.745, 4.270 vs. 2.833 in 5–8 F cells, *P* < 0.05). Ad-RAD50 combined with IR made a huge increased MTM relative to IR alone (38.086 vs. 8.461 in CNE1, *P* = 1.3E-17, 23.023 vs. 6.217 in 5–8 F cells, *P* = 3E-9).

### Ad-RAD50 infection increased radiosensitivity in vivo

**Fig. 7:**
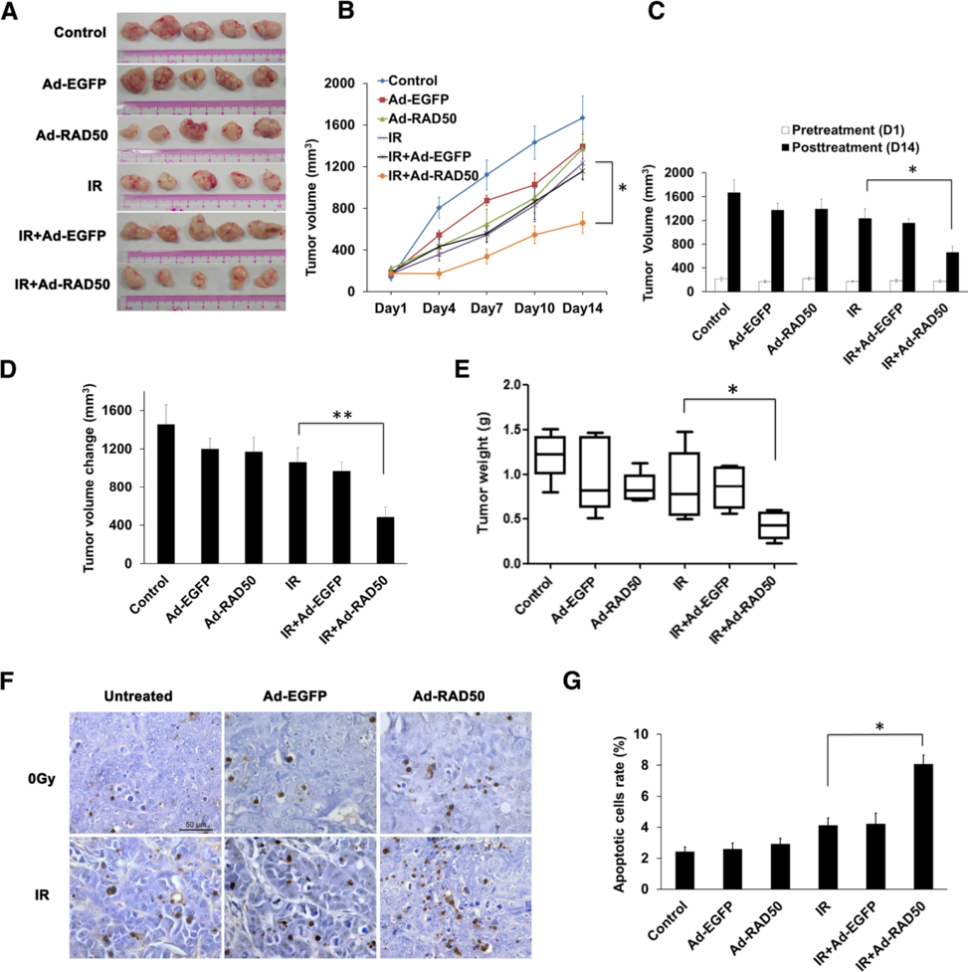
Effect of Ad-RAD50 infection on radiosensitivity of NPC xenografts in mice with IR treatment. **a** A photograph of a representative xenograft in each group when the mice were sacrificed 14 days after IR. **b** The tumor growth curve of CNE1 with different treatments. It was shown that IR combined with Ad-RAD50 could inhibit the tumor growth, in contrast to the IR alone. **c** Mean internally measured tumor volume ± SEM immediately at the time of IR treatment on day 1 and animal sacrificed on day 14. **d** Mean internally measured change in tumor volume ± SEM in each group. **e** The weights of tumors from xenografts on day 14 (Mean ± SEM). Ad-RAD50 infection combined with IR significantly decreased tumor weight compared with IR alone or IR combined with Ad-EGFP. **f** Tumor nodules were subjected to TUNEL assays. **g** The quantitative analysis of TUNEL assay. Ad-RAD50 infection combined with IR significantly increased the number of apoptotic cells, when compared with IR alone or IR combined with Ad-EGFP. Scale bar: 50 μm; *n* = 3; Values are depicted as the Mean ± SEM; *P <0.05 and **P < 0.01

Control group tumors exhibited sustained growth following cancer cell injection (Fig. 7-a, -b, Additional file 2: Figure S2), between days 1 and 14, IR combined with Ad-RAD50 infection significantly regressed the tumor growth compared with IR alone (*P* = 0.018). Control showed a 6.8 fold increase in tumor volume relative to pretreatment size. By contrast, Ad-EGFP group tumors showed 7.0 fold increases in tumor volume (*P* > 0.05) and a single intratumoral injection of Ad-RAD50 induced a significant suppression of tumor growth relative to controls, with 5.23 fold increase in tumor volume (*P* > 0.05, Fig. 7-c). Contrast to the IR alone (6.17 fold increase) or combined with Ad-EGFP treatment (5.23 fold increase), IR combined with Ad-RAD50 limited tumor growth (the tumor only acquired 2.73 fold crease, *P* = 0.011). As Fig. 7-d shown, the volumes of tumors (the mean volume change is 484.1 mm^3^) from mice treated with Ad-RAD50 combined with IR obtained the lowest growth when compared with the treatment IR alone (the mean volume change is 1061.9 mm^3^, *P* = 0.015) or combined with Ad-EGFP (the mean volume change is 969.9 mm^3^, *P* = 0.008). The mean weight (0.424 g) of tumors from mice treated with Ad-RAD50 combined with IR was decreased compared with mice treated with IR alone (mean weight is 0.868 g, *P* = 0.046) or Ad-EGFP combined with IR (mean weight is 0.754 g, *P* = 0.090) (Fig. 7-e). There were no significant difference among the weights of tumors from mice treated with Ad-RAD50, Ad-EGFP, IR alone and control. Besides, there was no significant difference in weight between treatment groups and controls (Additional file 3: Figure S3). No mice died during the experiment caused by treatments. This suggests mutant RAD50 gene transfer, with or without IR, is well tolerated.

TUNEL staining was used on tumor frozen sections, and the percentage of apoptotic cells was determined (Fig. 7-f and -g). Nontreated controls had low levels of apoptosis per high-powered field (2.425 %), by contrast, IR induced significantly more apoptosis, alone and combined with Ad-EGFP (4.127 % and 4.227 %, respectively; *P* > 0.05 for both). Combining Ad-RAD50 with IR markedly increased the amount of apoptosis in mice (8.071 %) with respect to either treatment with IR alone or combined with Ad-EGFP (*P* < 0.001).

## Discussion

Cancer cells can activate a series of pathways to repair DSBs and to maintain proliferation, all of which might significantly underlie radioresistance and tumor recurrence. It is apparent that an efficient DSBs repair response may be a hallmark of radioresistance.

Previous studies investigating DNA damage-signaling pathways have revealed that the MRN complex plays central roles in DNA repair and cell cycle checkpoints, and these molecules represent promising targets for radiosensitization [18]. The treatment with Mre11 siRNA increases human tumor cells radiosensitivity. The expression of full-length Nbs1 protein markedly enhances the radiosensitivity of the HNSCC cell line JHU011 [9, 19]. Besides, dysfunction of Rad50 directly reduces MRN complex-mediated DNA repair [20]. It was demonstrated that the phospho-site-specific (S635G) Rad50 mutant abrogates its activating phosphorylation by ATM, and Rad50-deficient cells exhibited defective DNA damage-induced signaling in cell cycle control and DNA repair [21]. In current study, we found that Rad50 was upregulated in irradiated NPC cells, which confirmed that Rad50 was a crucial factor of MRN complex function in NPC cells.

In order to enhance radiosensitivity by perturbing the DSBs repair capacity of NPC cells, we used a recombinant adenovirus containing a mutant Rad50 gene that consists only of the zinc hook region. Our previous study demonstrated that the infection of this recombinant adenovirus would specifically impair the DNA repair function of the MRN complex in head and neck tumor cells [17]. In present study, co-confocal and coimmunoprecipitation data confirmed that the mutant-Rad50 retained the ability to interact with the wild-type Rad50 via its zinc hook region but lost the ability to bind to Mre11. As a result, functional Rad50_2_/Mre11_2_ heterotetramers could not be assembled proximal to this mutant-Rad50, unlike wild-type Rad50, protein assembly could not coordinate around the mutant RAD50 protein, and a fully functional MRN octamer did not form. Moreover, the disruption of wild-type Rad50 by infection with the mutant-Rad50 repressed the levels of wild-type Rad50, Mre11 and Nbs1. This would be induced by a dominant-negative effect that disrupts the MRN complex. This dominant-negative effect has also been previously demonstrated through the use of RNA interference. Both transient and stable knockdown of the wild-type Rad50 gene induced a concurrent suppression in Mre11 and Nbs1 protein levels [22, 23].

The dysfunction of MRN would sensitize cells to DNA-damaging agents [16, 21]. It was proved by our comet assay results that NPC cells infected with Ad-RAD50 exhibited defective DNA repair. Between the NPC cells that were treated with or without IR, the mutant Rad50-infected cells that were treated with IR exhibited remarkable levels of DNA damage and the highest MTM values. Failure of proper DSBs repair would initial apoptosis, and MRN complex deficiency induced apoptosis by activating ATM and the corresponding downstream pathway [24, 25]. Besides, MRN related DSBs repair responses also activate DSBs induced cell cycle arrest. ATM activation induced by MRN complex promotes cell-cycle checkpoint induction; subsequently phosphorylate downstream cdc25c and cdk1/cyclinB complex to induce G2/M arrest [26]. G2/M arrest abrogate would enhance the tumor cells sensitivity of IR. In Wand *et al.* study, c-MYC (MYC) regulates radiotolerance in NPC through transcriptional activation of CHK1 (CHEK1) and CHK2 (CHEK2) checkpoint kinases through direct binding to the CHK1 and CHK2 promoters. Inhibition of MYC leads to the inactivation of CHK1/CHK2 pathway, eliminates DSBs-induced G2/M arrest, and subsequently promotes apoptosis and thus sensitizes NPC cells to IR [27]. The CHK1 inhibitor, Go6976 enhances the radiosenstivity is also associated the G2/M arrest abrogate [28]. In this study, we observed that Ad-RAD50 infection decreased the phosphorylation of cdc25c and cdk1. It was implied that the enhanced sensitivity of NPC cells to IR via Ad-RAD50 infection is also associated with abrogating DSBs induced G2/M arrest.

In addition to initialing DSBs repair, MRN complex might be involved in the recruitment or activity of telomerase or the maintenance of the telomeres, thus preventing chromosome ends from being recognized as DSBs [18]. Wild-type Rad50 was found to be a negative regulator of telomere maintenance that downregulates TRF1. Nbs1 downregulates TRF2 and contributes to telomere maintenance [29, 30]. As a positive regulator of telomere maintenance, the MRN complex induces TRF phosphorylation by ATM, triggering the release of TRF1 from telomeres and promoting telomerase access to the ends of telomeres [29]. Nbs1 was found to negatively regulate telomere length, resulting in accelerated telomere shortening in NBS cells [30]. Another mechanism by which MRN regulates telomere length is the form of recombination-mediated DNA replication known as alternative lengthening of telomeres (ALT) [23]. Kavitha *et al*. found that different cancer cells exhibit differential expression of MRN components and that targeting MRN complex subunits would affect the expression of the other MRN subunits, thus sensitizing a subset of cancer cells to radio- and/or chemotherapy [31]. In this study, the expression of mutant Rad50 disrupted the function of wild-type Rad50, abrogating proper MRN complex function. Our data suggested that infection with Ad-RAD50 increases the sensitivity of NPC cells to IR, likely by shortening the length of their telomeres. The same sensitization to IR has also been reported in other cancers, such as head and neck cancer [9].

In all, Ad-RAD50 would enhance DSBs induced by IR, abrogate G2/M arrest and thus reduce the DSBs repair time, and probably impact maintenance of the telomeres to prevent DSBs recognition via disturbing MRN complex functions, Ad-RAD50 would increase the sensitivity of NPC cells to IR. It was confirmed by that mutant RAD50 expressed, MRN-deficient cells exhibited cell growth inhibition by MTT assay in vitro, and by the colony formation assay that Ad-RAD50 infection brought out obviously decrease in NPC cells survival fraction after IR. Moreover, Ad-RAD50 combined with IR produced a dramatic tumor regression in human NPC xenografts.

This is the first report to our knowledge translating a RAD50-disrupting approach to antitumor therapy in vitro and in NPC xenografts. Our findings represent a novel strategy for increasing the radiosensitivity of NPC in patients.

## Conclusions

This study for the first time provides insight into a new therapeutic approach to NPC radiosensitization via targeted native cellular RAD50 disruption by expressing a mutant rad50 only containing Rad50 zinc hook domain but lacking the ATPase domain and the Mre11 interaction domain. This mutant rad50 expression would disrupt native cellular MRN functions in abrogating DSBs induced G2/M arrest, increasing DSBs induced by irradiation and apoptosis, and finally sensitize NPC to IR in vitro and in vivo.

## Additional files

Additional file 1: Figure S1. Ad-RAD50 enhanced IR-induced DNA damage in CNE1 cells at different times. (A), (B) and (C) show the DNA DSBs in CNE1 tumor cells as detected by neutral comet assay at 0, 12 and 24 h after IR treatment, respectively. (D), (E) and (F) Damaged and fragmented DNA migrate toward the anode, producing a comet tail. Original magnification: ×40; the mean olive tail movements (MTMs), which were measured in CNE1 at 0, 12 and 24 h, respectively, after IR treatment using CASP software. MTM values ± SEM are shown. Ad-RAD50 combined with 4Gy IR significantly increased DSBs compared to all the other groups (*P* < 0.001). * *P* < 0.05, ** *P* < 0.01. (TIF 3488 kb) Additional file 2: Figure S2. NPC xenografts of mice with 6 different treatments. (TIF 7044 kb) Additional file 3: Figure S3. The final weights of mice with 6 different treatments. (TIF 537 kb)

## Abbreviations

ABC domain: The bipartite ATP-binding cassette domain
Ad-EGFP: Empty control adenovirus expressing EGFP protein
Ad-RAD50: A recombinant adenovirus containing a mutant Rad50 gene
DSBs: DNA double strand breaks
HR: Homologous recombination
hRAD50: Human RAD50 gene
IR: Irradiation
MOI: Multiplicity of infection
MRN: Mre11-Rad50-Nbs1
MTM: The median tail moments
MTT: 3-(4,5-dimethyl-2-thiazolyl)-2,5-diphenyl-2-H-tetrazolium bromide
NHEJ: Non-homologous end joining
NPC: Nasopharyngeal cancer
OS: Overall survival
PFUs: Plaque forming units
