# Supplementary figures and images for: Targeting Rad50 sensitizes human nasopharyngeal carcinoma cells to radiotherapy
Source: BMC Cancer. 2016 Mar 7;16:190. doi: 10.1186/s12885-016-2190-8 (PMC4782334; doi:10.1186/s12885-016-2190-8)

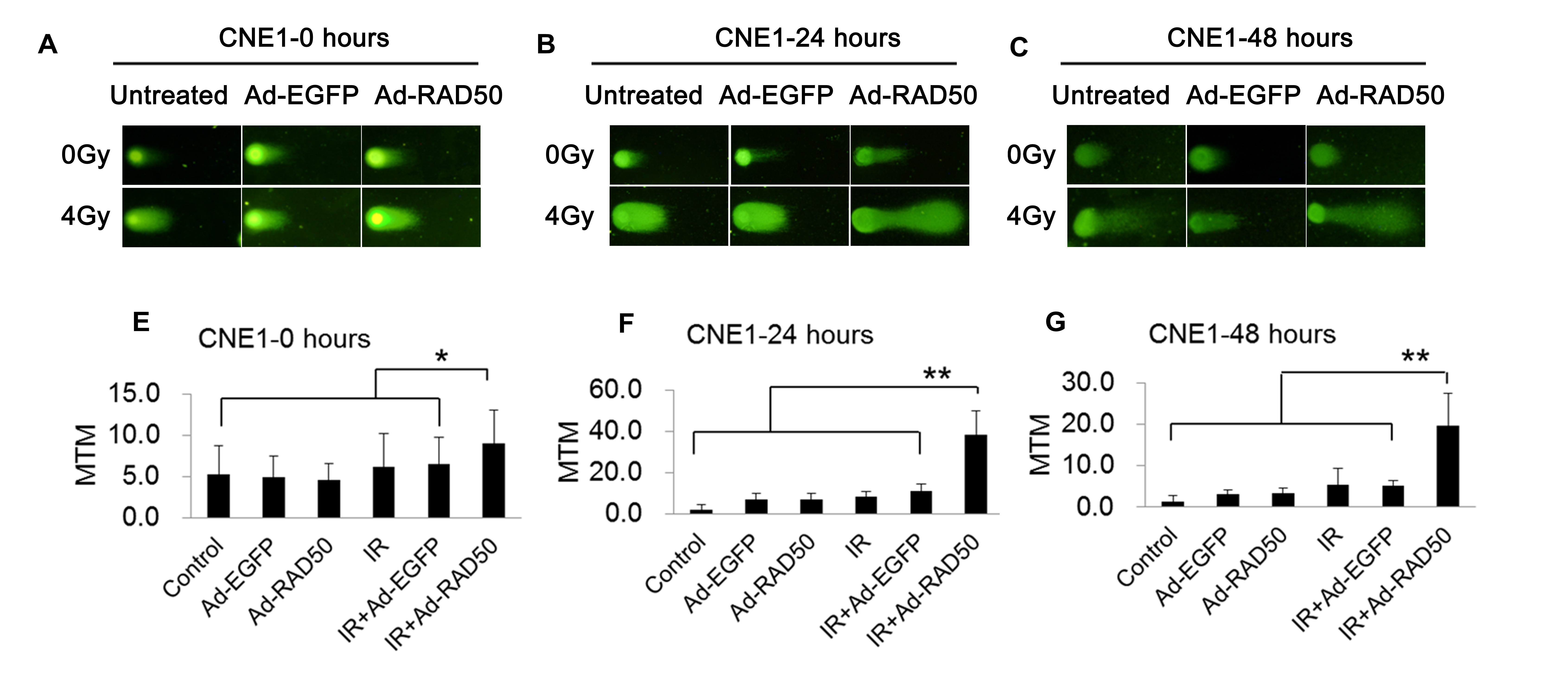

Supplement: Additional file 1: Figure S1. — Ad-RAD50 enhanced IR-induced DNA damage in CNE1 cells at different times. (A), (B) and (C) show the DNA DSBs in CNE1 tumor cells as detected by neutral comet assay at 0, 12 and 24 h after IR treatment, respectively. (D), (E) and (F) Damaged and fragmented DNA migrate toward the anode, producing a comet tail. Original magnification: ×40; the mean olive tail movements (MTMs), which were measured in CNE1 at 0, 12 and 24 h, respectively, after IR treatment using CASP software. MTM values ± SEM are shown. Ad-RAD50 combined with 4Gy IR significantly increased DSBs compared to all the other groups (P < 0.001). * P < 0.05, ** P < 0.01. (TIF 3488 kb) [file 12885_2016_2190_MOESM1_ESM.tif]

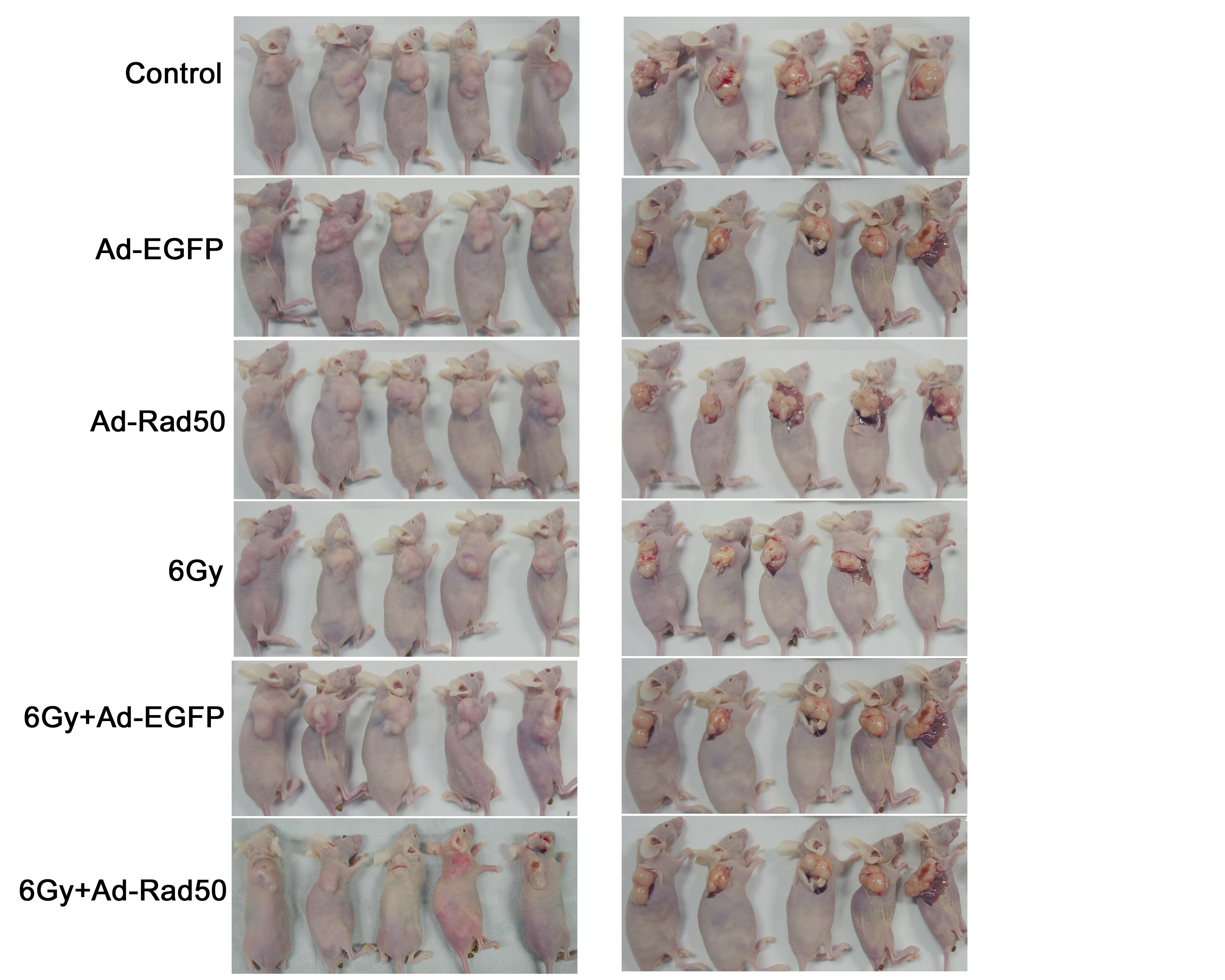

Supplement: Additional file 2: Figure S2. — NPC xenografts of mice with 6 different treatments. (TIF 7044 kb) [file 12885_2016_2190_MOESM2_ESM.tif]

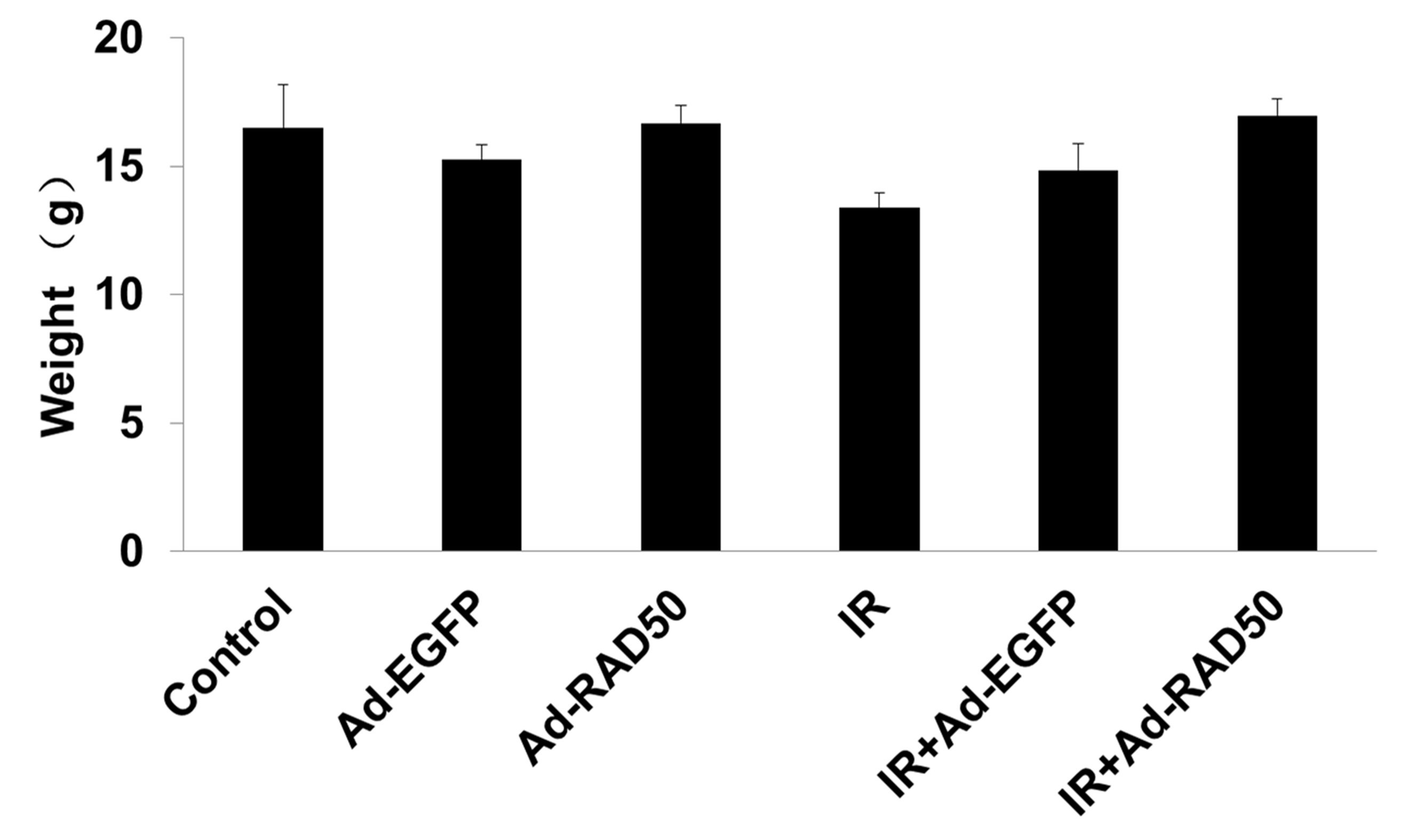

Supplement: Additional file 3: Figure S3. — The final weights of mice with 6 different treatments. (TIF 537 kb) [file 12885_2016_2190_MOESM3_ESM.tif]
